# Bidirectional Optogenetic Modulation of the Subthalamic Nucleus in a Rodent Model of Parkinson’s Disease

**DOI:** 10.3389/fnins.2022.848821

**Published:** 2022-05-17

**Authors:** Caroline Xie, John Power, Asheeta A. Prasad

**Affiliations:** ^1^School of Psychology, University of New South Wales, Sydney, NSW, Australia; ^2^Department of Physiology and Translational Neuroscience Facility, School of Medical Sciences, University of New South Wales, Sydney, NSW, Australia; ^3^Faculty of Medicine and Health, School of Medical Sciences, University of Sydney, Sydney, NSW, Australia

**Keywords:** Parkinson’s disease, subthalamic nucleus, optogenetics, 6-hydroxydopamine, motor symptoms

## Abstract

Parkinson’s disease (PD) is a neurodegenerative disorder characterized by a range of motor symptoms. Treatments are focused on dopamine replacement therapy or deep brain stimulation (DBS). The subthalamic nucleus (STN) is a common target for DBS treatment of PD. However, the function of the STN in normal conditions and pathology is poorly understood. Here, we show in rats that optogenetic modulation of STN neuronal activity exerts bidirectional control of motor function, where inhibition of the STN increases movement and STN activation decreases movement. We also examined the effect of bidirectional optogenetic manipulation STN neuronal activity under dopamine depleted condition using the bilateral rodent 6-hydroxydopamine (6-OHDA) model of Parkinson’s disease. Optogenetic inhibition of the STN in the absence of dopamine had no impact on motor control yet STN excitation led to pronounced abnormal involuntary movement. Administration of levodopa rescued the abnormal involuntary movements induced by STN excitation. Although dopamine and STN dysfunction are well established in PD pathology, here we demonstrate simultaneous STN over activity and loss of dopamine lead to motor deficits. Moreover, we show the dysfunction of the STN is dependent on dopamine. This study provides evidence that the loss of dopamine and the over activity of the STN are key features of PD motor deficits. These results provide insight into the STN pathology in PD and therapeutic mechanism of targeting the STN for the treatment for PD.

## Highlights

- Our study shows direct role of STN in bidirectional regulation of motor control, where increase in STN activity decreases movement and inhibition of STN increases movement.
- Motor functions regulated by STN are dependent on dopamine.
- Administration of levodopa rescued the motor deficits induced by STN activation in the neurotoxin PD rodent model.
- The combination of the traditionally applied neurotoxin rodent model with optogenetics provides relevant rodent model for assessing pharmacotherapies for PD motor symptoms.

## Introduction

Parkinson’s disease (PD) is a complex neurological disorder with pathological hallmarks of dopamine loss and hyperactivity of the subthalamic nucleus (STN) (Levy et al., 2000). Links between STN and PD were clearly demonstrated in a non-human primate model of PD study where motor deficits were reversed by STN lesions (Bergman et al., 1994). Moreover, deep brain stimulation (DBS) of the STN has been a successful treatment for PD patients for over 30 years (Limousin and Foltynie, 2019). Interestingly, post-mortem human brain studies in PD cases show no significant changes in the number of neurons in the STN (Hardman et al., 1997). The primary pathology in the clinical studies have found increased firing rates of the STN in PD patient’s correlating with severity of motor deficits (Levy et al., 2000).

The STN was anatomically identified in humans by Jules Bernard Luys (Parent, 2002). Its distinctive biconvex morphology is shared across humans and rodents (Hardman et al., 1997; Alkemade et al., 2019; Wallén-Mackenzie et al., 2020). STN is part of the basal ganglia and executes motor function as part of the indirect striatal pathway with inputs from the globus pallidus or the hyper direct pathway with inputs from the cerebral cortex (Gerfen, 1992). The emergence of optogenetic technologies to stimulate and inhibit specific neuronal populations with high temporal precision has revolutionised our understanding of striatal pathways components. For example, temporal optogenetic activation of striatal D2 receptor expressing neurons (indirect pathway) decreases movement (Kravitz et al., 2010). Activation of parvalbumin terminals in the STN arising from the GP and direct optogenetic inhibition of STN neurons promotes movement (Pamukcu et al., 2020). These optogenetic studies provide insight in specific neurons and pathways in motor regulation.

However, the effect of DBS on individual components of the striatal pathways in pathology remains unclear. In the absence of dopamine, other pre-clinical PD studies have activated striatal neurons to induce dyskinesia (Kravitz et al., 2010; Hernández et al., 2017). However, direct optogenetic inhibition of STN neurons in the unilateral neurotoxin rodent PD model had no effect on motor control (dyskinesia); instead motor dysfunction was rescued by activation of the hyper-direct pathway (Gradinaru et al., 2009). In clinical cases, pathologies of the STN and dopamine loss co-exist it is difficult to determine if one precedes the other and their independent contributions in motor dysfunction. We hypothesise that STN dysfunction is dependent on dopamine. To address the contribution of STN functions in motor control in normal and PD motor pathology, here we examined both STN activation and inhibition in normal and dopamine lesion rats.

## Materials and Methods

### Subjects

Adult male Long Evans (Randwick, UNSW) weighing between 300 and 350 g at the time of surgery. Rats were housed in a colony room maintained on 12:12 h light/dark cycle (lights on at 7.00 a.m.) in ventilated racks in groups of four. Food and water were available *ad libitum*. All procedures were approved by the Animal Care and Ethics Committee at The University of New South Wales and conducted in accordance with the NIH Guide for the Care and Use of Laboratory Animals. The procedures were designed to minimise the number of animals used.

### Surgeries

Stereotaxic surgeries was done as previously described in Prasad et al. (2020). Briefly, rats were anesthetized *via* intraperitoneal injection with a mixture of 1.3 ml/kg ketamine anesthetic (Ketapex; Apex Laboratories, Sydney, Australia) at a concentration of 100 mg/ml and 0.3 ml/kg of the muscle relaxant xylazine (Rompun; Bayer, Sydney, Australia) at a concentration of 20 mg/ml. Rats received a subcutaneous injection of 0.1 ml 50 mg/ml carprofen (Pfizer, Tadworth, United Kingdom) before being placed in the stereotaxic frame (Kopf Instruments, Tujunga, CA, United States). The stereotaxic coordinates were based on a rat brain atlas (Paxinos and Watson, 2009), following flat skull coordinates relative to bregma in mm: (AP, ML, DV): STN (−3.6 AP, ±2.6 ML, −8.0 DV), STN optic fibers (−3.6 AP, ±2.6 ML, −7.5 DV), and striatum (+1.28 AP, ±3.0 ML, −5.2 DV). Optic cannulas were secured using jeweller’s screws and dental cement (Vertex Dental, Netherlands). Viral vectors or 6-OHDA were infused with a 23-gauge, cone-tipped 5 μl stainless steel injector (SGE Analytical Science) using an infusion pump (UMP3 with SYS4 Micro-controller, World Precision Instruments, Inc., Sarasota, FL, United States). The needle was left in place for 10 min to allow for diffusion and reduce spread up the infusion tract. At the end of surgery, rats received intramuscular injection of 0.2 ml of 150 mg/ml solution of procaine penicillin (Benacillin; Troy Laboratories, NSW, Australia) and 0.2 ml of 100 mg/ml cephazolin sodium (AFT Pharmaceuticals, North Ryde, NSW, Australia).

### Viral Vectors

AAV5-CaMKII-eYFP, AAV5-CaMKII-HA-eNpHR3.0-IRES-Eyfp, or AAV5-CaMKII-HA-eChR2-IRES-eYFP at a minimum of 2 × 10^12^ viral particles per ml were obtained from the UNC Vector Core, University of North Carolina. Vector volume of 300 nl (150 nl/min) was infused per hemisphere.

### Drugs

6-OHDA (6-Hydroxydopamine hydrobromide; H116, Sigma-Aldrich, Australia) was dissolved with 0.02% ascorbic acid saline solution (3 μg/μl). Rats either received 12 μg (4 ul of 3 μg/μl 6-OHDA) unilaterally into the medial forebrain bundle (MFB) or 6 μg (2 ul of 3 μg/μl 6-OHDA) bilaterally into the striatum. L-dopa (25 mg/kg, methyl L-dopa hydrochloride; #D1507, Sigma-Aldrich) and benserazide (10 mg/kg, benserazide hydrochloride; # B0477000, Sigma-Aldrich) (1 ml/kg; i.*p*.), 20 min prior to test session similar to Moreira Vasconcelos et al. (2020).

### Optogenetic Manipulation

LEDs and optical parameters were as previous applied in Gibson et al. (2018). Fibre optic cannulae and patch cables were fabricated from 0.39 NA, Ø400 μm core multimode optical fiber and ceramic ferrules (Thor Labs, Newton, NJ, United States). Optic cannulas on animals were attached to patch cables connected to 625 nm LEDs (Doric Lenses Inc., Quebec, Canada) or 470 nm LEDs (Doric Lenses Inc., Quebec, Canada). 625 nm light was delivered as continuous illumination (8–10 mW) and 470 nm light was delivered as trains of 20 ms light pulses (10–12 mW, 12.5 Hz) (Gibson et al., 2018).

### Electrophysiological Validation of Opsins Activity

Brain slices were prepared from rats that had received (AAV5-CaMKII-HA-eNpHR3.0-IRES-eYFP or AAV5-CaMKII-HA-ChR2-IRES-eYFP) into the STN (−3.6 AP, ±2.6 ML, −8 DV) at least 6 weeks before slice preparation. Rats were deeply anesthetized with isoflurane (5%), decapitated and their brain rapidly removed and submerged in ice-cold oxygenated (95% O_2_, 5%CO_2_) HEPES based artificial cerebral spinal fluid [HEPES-aCSF; (in mM) 95 NaCl, 2.5 KCl, 30 NaHCO_3_, 1.2 NaH_2_PO_4_, 20 HEPES, 25 glucose, 5 ascorbate, 2 thiourea, 3 sodium pyruvate, pH adjusted to 7.3–7.4 with NaOH] with low (0.5 mM) CaCl_2_, and high (10 mM) MgSO4 for 2–3 min. Coronal slices (300 μm) were made using a vibratome (model VT1200, Leica) and then incubated for 10 min in a 30°C neural protective recovery HEPES-aCSF (NaCl was replaced by equimolar N-methyl-D-glucamine, pH adjusted to 7.3–7.4 with HCl), and then transferred to a Braincubator (Payo Scientific, #BR26021976) and maintained at 16°C in a HEPES-aCSF holding solution with 2 mM CaCl_2_, and 2 mM MgSO_4_.

For recordings, slices were transferred to a recording chamber and continuously perfused with standard aCSF (30°C) containing (in mM); NaCl, 124; KCl, 3; NaHCO_3_, 26; NaH_2_PO_4_, 1.2; glucose, 10; CaCl_2_, 2.5; and MgCl_2_, 1.3. Targeted whole-cell patch-clamp recordings were made from eYFP+ STN neurons using a microscope (Zeiss Axio Examiner D1) equipped with 20× water immersion objective (1.0 NA), LED fluorescence illumination system (pE-2, CoolLED) and an EMCCD camera (iXon+, Andor Technology). Patch pipettes (3–5 MΩ) were filled with an internal solution containing 130 mM potassium gluconate, 10 mM KCl, 10 mM HEPES, 4 mM Mg_2_-ATP, 0.3 mM Na_3_-GTP, 0.3 mM EGTA, and 10 mM phosphocreatine disodium salt (pH 7.3 with KOH, 280–290 mOsm). Electrophysiological recordings were amplified using a Multiclamp amplifier (700B, Molecular Devices, CA, United States), filtered at 6–10 kHz, and digitized at 20 kHz with a National Instruments multifunction I/O device (PCI-6221). Recordings were controlled and analysed offline using Axograph (Axograph, Sydney, Australia).

Electrophysiological data were analysed off-line using AxoGraph. Series resistance, membrane resistance, and cell capacitance were calculated using in built routines in Axograph. ChR2 and eNpHR3.0 were stimulated using blue (470 nm) and orange light (GYR LED bandpass filtered 605/50 nm) delivered through the objective. When possible, protocols were repeated up to five times and the results averaged. Data were excluded if the series resistance was >25 MΩ or more than 100 pA was required to maintain the neuron at -60 mV. Liquid junction potentials were uncompensated.

## Behavioural Procedures

### Open Field

Locomotor activity was assessed in Plexiglas chambers (Med Associates; width = 43.2 cm, length = 43.2 cm, height = 30.5 cm). Movement was tracked with three 16 beam infrared arrays. Infrared beams were located on both the x- and y-axes for positional tracking of multiple motor behaviours including ambulatory distance, episodes, counts, stereotypic, resting, and vertical time. Suspended above the chamber was a camera and LED plus fiber-optic rotary joint controlled by LED driver (Doric Lenses). There were three equal segments for test sessions, a pre-stimulation period followed by optic stimulation (stimulation period) and post-stimulation. In non-lesioned rats, Figure 2 each segment was 1 min and the case of lesioned rats each phase was reduced to 10 s to minimise the duration of severe motor symptoms. The total distance travelled and average speed were recorded using the tracking system.

### Real Time Place Preference Test

Real time place preference test was assessed in a custom-made behavioural arena consisting of two plexiglass chambers (50 × 50 × 50 cm) using a protocol adapted from Stamatakis and Stuber (2012). One chamber was assigned as the stimulation side and the other non-stimulated, which was counter balanced. The rat was placed in the non-stimulated side at the onset of the experiment and each time the rat crossed to the stimulation side of the chamber, optogenetic stimulation was delivered until the rat crossed back into the non-stimulation side. The session was for 20 min and video recorded. The time spent in each chamber was scored manually.

### Abnormal Involuntary Movements Scoring

Abnormal involuntary movements (AIMs) were scored according to a rat dyskinesia scale (Cenci and Lundblad, 2007). Stereotypic behaviour was classified into three subtypes; axial dystonia, limb dyskinesia, and orolingual dyskinesia. During optical stimulation, rats were assessed for each subtypes on a scale (0–4) of progressive severity. The total AIMs score for each rat was calculated by adding the individual scores of each subtype of stereotypic behaviour, hence the maximum score a rat could obtain would be 12.

### Bidirectional Regulation of Motor Function With Optogenetic Manipulation of the Subthalamic Nucleus

Rats were assigned to three groups, eYFP, ChR2, and NpHR3.0, which received AAV5-CaMKII-eYFP, AAV5-CaMKII-HA-ChR2-IRES-eYFP, and AAV5-CaMKII-HA-eNpHR3.0-IRES-eYFP bilaterally in the STN, respectively. Optic cannulas were placed 0.5 mm above the STN right above the viral injection. After a minimum of 3 weeks post-surgery, all groups test assessed for locomotor and real time place preference test during optogenetics manipulation.

### Bidirectional Regulation of Subthalamic Nucleus the Bilateral 6-Hydroxydopamine Model

There were four groups; eYFP +Saline, eYFP +6-OHDA, ChR2 +6-OHDA, and NpHR3.0 +6-OHDA. All rats received bilateral injections of either saline or 6-OHDA in the striatum, followed by bilateral viral injections in the STN with AAV5-CaMKII-eYFP, AAV5-CaMKII-HA-eNpHR3.0-IRES-eYFP or AAV5-CaMKII-HA-eChR2-IRES-eYFP. Optic fibers were secured above the STN. After a minimum of 3 weeks post-surgery, all groups were assessed for motor behaviour during optogenetic manipulation.

### Immunohistochemistry

At the conclusion of the experiments, rats were deeply anesthetized with sodium pentobarbital (100 mg/kg, i.*p*.) and perfused transcardially with 150 ml of 0.9% saline, containing heparin (5000 i.u/ml), followed by 400 ml of 4% paraformaldehyde in 0.1 M phosphate buffer (PB), pH 7.4. Brains were post-fixed for 1 h in the same fixative and placed in 20% sucrose solution overnight. Brains were frozen and sliced to 40 μm coronal sections using Leica CM3050 cryostat. Four serially adjacent sets from the STN and substantia nigra were obtained from each brain and stored in 0.1% sodium azide in 0.1 M PBS, pH 7.2 to assess level of dopamine loss in the SN and virus expression and cannuale placement.

To detect tyrosine hydroxylase (TH) expression, sections were washed in 0.1 M PB, followed by 50% ethanol, 50% ethanol with 3% hydrogen peroxidase, then 5% normal horse serum (NHS) in PB (30 min each). Sections were then incubated in sheep antiserum against TH (1:2000; cat. no. AB1542, Life Technologies) in a PB solution blocking buffer (2% NHS and 0.2% Triton X-10) (48 h at 4°C). The sections were then washed and incubated in biotinylated donkey anti-goat (1:1000; Jackson ImmunoResearch Laboratories) for 24 h at 4°C. Finally, the sections were incubated in avidin-biotinylated horseradish peroxidase complex (Vector Elite kit: 6 μl/ml avidin and 6 μl/ml biotin; Vector Laboratories, 2 h at room temperature), washed in PB, and then incubated (15 min) in a diaminobenzidine solution (DAB) containing 0.1% 3,3-diaminobenzidine, 0.8% D-glucose and 0.016% ammonium chloride. Immunoreactivity was catalysed by the addition of 0.2 μl/ml glucose oxidase (24 mg/ml, 307 U/mg, Sigma). Brain sections were then washed in PB pH 7.4. Sections were mounted onto gelatin-coated slides, dehydrated, cleared in histolene, and cover-slipped with Entellan (Proscitech). To quantify the extent of dopamine depletion, manual counts of TH-immunoreactive neurons were made using Adobe Photoshop software. For each rat, counting was restricted to SN of the coronal section that best matched bregma -5.20 to -5.55 of the rat brain atlas (Paxinos and Watson, 2009). Cell counts were performed blind to the experimental group.

For the detection of viral vector expression, brain sections were washed in 0.1 M PB, then 5% normal horse serum (NHS) in PB (30 min each) and then incubated in rabbit antiserum against eGFP (1:2000; cat. no. AA11122, Life Technologies) for 24 h at 4°C. The primary antibodies were diluted in blocking buffer. After washing off unbound primary antibody, sections were incubated overnight at 4°C in AlexaFluor-488 conjugate (1:1000, A11034, Invitrogen). Brain sections were then washed in PB, pH 7.4, and mounted using mounting media. eGFP-immunoreactivity was assessed to determine the extent of transfection and optic cannula placements were imaged using Olympus light microscope (BX53) at 10× magnification.

### Data Analyses

Data in figures and table are represented as mean ± SEM. The criteria for inclusion in final analysis was correct AAV or tracer and/or fiber placements determined after histology. Group numbers used for analyses in each experiment are indicated under the subheadings of behavioural procedures above. Our primary behavioural dependent variables were locomotor activity. A one-way ANOVA with Bonferroni *post-hoc* test was used to compare group differences. A paired samples *t*-test was used to compare means pre and post Levodopa administration. Statistical significance was set as *p* < 0.05. All statistical procedures were performed with SPSS 27 and GraphPad Prism 7.

## Results

### Experiment 1: Electrophysiological Validation of Opsins in the Subthalamic Nucleus

**FIGURE 1:**
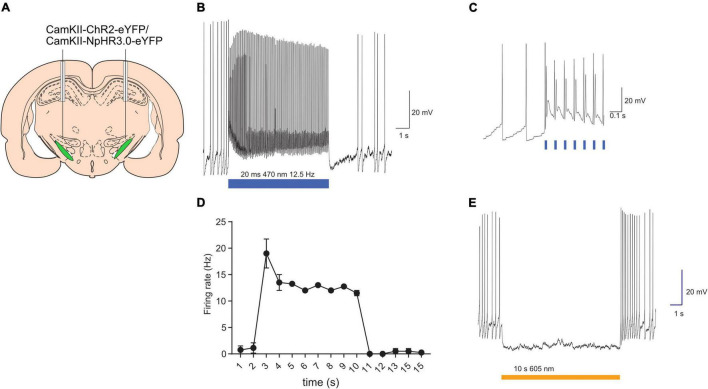
Validation of opsins activity with *in vitro* electrophysiology. Schematic of subthalamic nucleus targeting of opsins **(A)**. Example of light-evoked excitation of ChR2-expressing neurons **(B)**. Light-evoked excitation shown on an expanded timescale to revealing synchrony between light onset and action potential generation **(C)**. Summary data plotting mean (±SEM; *n* = 4) firing frequency in presence and absence of light stimulation **(D)**. Example of light-evoked suppression of neuronal firing in eNpHR3.0-expressing neurons **(E)**. Bars indicate timing of light presentations. *p < 0.05.

The capacity of optogenetic manipulations to modulate STN neuronal firing was validated using *in vitro* electrophysiological recordings. Targeted whole-cell patch-clamp recordings were made from eYFP+ STN neurons in brain slice prepared from rats injected with AAV-CaMKII-ChR2-eYFP or AAV-CaMKII-eNpHR3.0-eYFP (Figure 1A). Majority of STN neurons are glutamatergic (Schweizer et al., 2014) and the CaMKII promotor has been used for selective expression opsin constructs STN glutamatergic neurons (Gradinaru et al., 2009; Wu et al., 2020). Consistent with previous reports these neurons had depolarized membrane potentials and often fired spontaneously (Bevan and Wilson, 1999). The membrane resistance and cell capacitance were 560 ± 362 MΩ and 7.8 ± 1.7 pF (mean ± SD; *n* = 6). ChR2 expressing neurons were stimulated with trains of brief (20 ms) blue light pulses (12.5 Hz 8 s). Each light pulse evoked a rapid depolarisation that evoked 1 or more action potentials (Figures 1B–D; *n* = 4 neurons/2 rats). Photo-stimulation of eNpHR3.0 expressing neurons evoked a rapid hyperpolarisation that reliably supressed neuronal firing for the duration of the light (Figure 1E; *n* = 2/2). Together, these results confirm our capacity to modulate STN neuronal activity with light.

### Experiment 2: Bidirectional Optogenetic Manipulation of the Subthalamic Nucleus Induces Opposing Effects on Locomotor Activity

**FIGURE 2:**
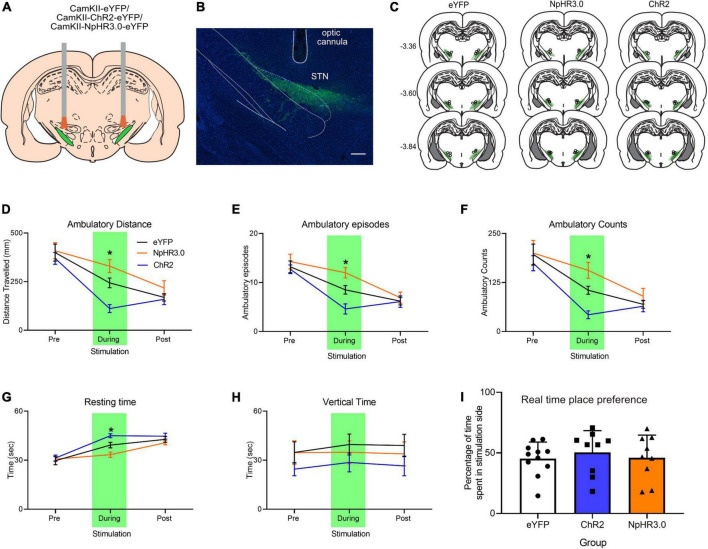
Bidirectional regulation of motor control with optogenetic manipulation of the subthalamic nucleus. **(A)** Schematic bidirectional optogenetic manipulation subthalamic nucleus. **(B)** Representative immuno-histological image of eYFP expression in the STN and optic cannula placement, scale bar: 250 mm. **(C)** Placement of viral expression and optic cannula in the STN. Total of 3 min open field test involving pre, during, and post-stimulation periods, 1 min per phase showing **(D)** ambulatory distance, **(E)** ambulatory episodes, **(F)** ambulatory counts, **(G)** resting time, and **(H)** vertical time. **(I)** Graph showing real-time place preference. Data are expressed as mean ± SEM. *p < 0.05.

There were three groups, eYFP (*n* = 10), ChR2 (*n* = 10), and NpHR3.0 (*n* = 11) that received microinjections of AAV-CaMKII-eYFP, AAV-CaMKII-ChR2-eYFP, and AAV-CaMKII-eNpHR3.0-eYFP, respectively, into the STN (Figures 2A–C). Light was delivered *via* an optical fiber inserted dorsal to the STN. Locomotor activity was measured pre-stimulation, stimulation, and post-stimulation in a single 3-min session (one minute per phase). Locomotion measurements for ambulatory distance travelled, ambulatory episodes ambulatory counts, resting time and vertical time were recorded by MedPC open field arena software. Mean and ±SEM are reported in Table 1. One-way ANOVA analysis showed no group differences for ambulatory distance during pre-stimulation, *F*_(2,28)_ = 0.264, *p* = 0.770 and post-stimulation phase, *F*_(2,28)_ = 1.152, *p* = 0.331. There were significant differences between groups only during stimulation phase, *F*_(2,28)_ = 16.210, *p* < 0.001. *Post hoc* analysis showed significant difference between groups. Locomotion measurements for ambulatory distance in eYFP group was higher than and ChR2, *p* = 0.002, eYFP was lower than NpHR3.0, *p* = 0.033, and NpHR3.0 group was higher than ChR2 group, *p* < 0.001 (Figure 2D).

**TABLE 1:** Mean ± SEM of ambulatory distance, ambulatory episodes, ambulatory counts, stereotypic time, resting time and vertical time during pre-stimulation, optogenetic stimulation and post-stimulation.

	Pre-stimulation						During stimulation						Post stimulation					
			
	eYFP		NpHR3.0		ChR2		eYFP		NpHR3.0		ChR2		eYFP		NpHR3.0		ChR2	
	Mean	±SEM	Mean	±SEM	Mean	±SEM	Mean	±SEM	Mean	±SEM	Mean	±SEM	Mean	±SEM	Mean	±SEM	Mean	±SEM
Ambulatory Distance	399.3	43.8	408.8	41.6	370	30.8	243.7	25.1	329.9	33.4*	111.6	20.6*	168.8	18.9	217.3	37	159.1	27.7
Ambulatory Episodes	13.2	1.2	14.3	1.5	12.7	0.9	8.5	0.9	12	1.1*	4.6	1.0*	6.2	0.8	6.9	1.1	6.1	1.2
Ambulatory Counts	196.7	26.4	200.4	31.8	174.3	19.1	105.3	10.3	156.2	20.1*	42.9	10.1*	69.4	10	90.1	20	64.5	14.2
Stereotypic Time	17	1.2	20.4	0.6	17.1	1.1	15.4	1.2	19.4	1.2	12.8	1.1	13.9	1.4	15.3	0.9	12.4	1.6
Resting Time	29.7	2.5	30.7	1.6	31.3	1.8	39.3	1.7	33.4	1.6*	45	1.2*	42.8	1.8	40.7	1.3	44.7	1.8
Vertical Time	34.8	6.5	34.6	7.3	24.5	4	39.6	6.4	34.9	7	28.6	5.8	39	6.9	33.9	7.4	26.5	6

* p < 0.05

Measurements for ambulatory episodes showed no group differences for during pre-stimulation, *F*_(2,28)_ = 0.425, *p* = 0.658 and post-stimulation phase, *F*_(2,28)_ = 0.179, *p* = 0.837. There were significant differences between groups only during stimulation phase, *F*_(2,28)_ = 13.900, *p* < 0.001. *Post hoc* analysis showed significant decrease between eYFP and ChR2, *p* = 0.011, increase between eYFP and NpHR3.0, *p* = 0.019 and increase between ChR2 and NpHR3.0, *p* < 0.001 (Figure 2E). For ambulatory counts, there was no difference between groups during pre-stimulation, *F*_(2,28)_ = 0.276, *p* = 0.761 and post-stimulation phase, *F*_(2,28)_ = 0.770, *p* = 0.473. There were significant differences between groups only during stimulation phase, *F*_(2,28)_ = 14.905, *p* < 0.001. *Post hoc* analysis showed significant decrease between eYFP and ChR2, *p* = 0.007, increase between eYFP and NpHR3.0, *p* = 0.021 and increase ChR2 and NpHR3.0, *p* < 0.001 (Figure 2F). Resting time was not different between groups during pre-stimulation *F*_(2,28)_ = 0.155, *p* = 0.857 and post-stimulation phase, *F*_(2,28)_ = 1.484, *p* = 0.244. There were significant differences between groups only during stimulation phase *F*_(2,28)_ = 14.346, *p* < 0.001. *Post hoc* analysis showed significant decrease between eYFP and ChR2, *p* = 0.015, and increase between eYFP and NpHR3.0, *p* = 0.011 (Figure 2G). Vertical time was not significantly different across all phases, *F*_(2,28)_ > 0.902, *p* > 0.503 (Figure 2H). Real time place preference was expressed as the percentage of time spent in stimulated chamber, mean ± SEM for eYFP was 45.37% ± 4.09, ChR2 was 45.32% ± 7.75 and NpHR3.0 was 46.08% ± 6.20 (Figure 2I). There was no significant difference between groups, *F*_(2,28)_ = 0.248, *p* = 0.782.

### Experiment 3: Bidirectional Optogenetic Manipulation of the Subthalamic Nucleus in Bilateral 6-Hydroxydopamine Lesioned Rats

We next examined the impact of optogenetic manipulation of STN neurons on motor symptoms in 6-OHDA preclinical model of PD. Dopamine neurons in the substantia nigra are most vulnerable in PD (Damier et al., 1999). Nigral neurons project to the dorsal striatum in contrast to the dopamine neurons in the ventral tegmental area which project mainly to the ventral striatum (Prensa and Parent, 2001). Lesions of the dorsolateral striatum contribute to motor regulation (Pisa, 1988). Hence, we injected 6-OHDA into the dorsal lateral region of the striatum to preferentially lesion nigral dopamine neurons.

**FIGURE 3:**
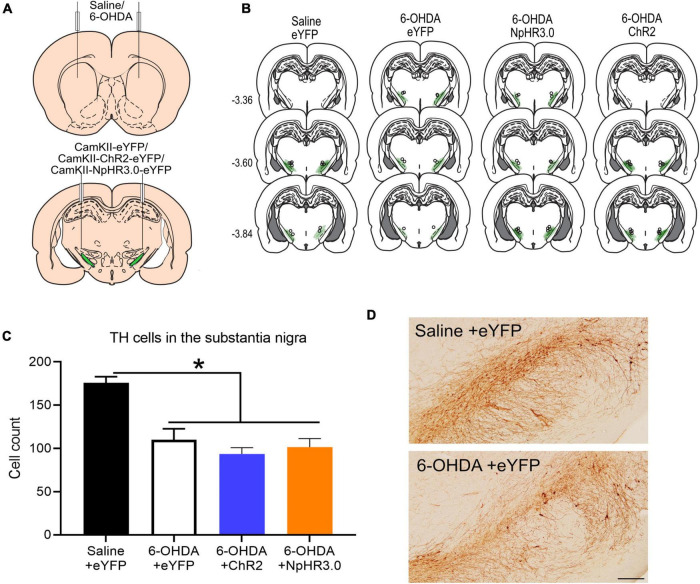
Bidirectional regulation of subthalamic nucleus in dopamine in PD rodent model: Schematic of bilateral saline/6-OHDA administration in the striatum and optogenetic manipulation subthalamic nucleus **(A)**. Placement of viral expression and optic cannula in the STN **(B)**. Graph of tyrosine hydroxylase (TH)-immunoreactive cells in the substantia nigra in all groups **(C)**. Representative TH immunostaining from coronal sections of the substantia nigra in saline +eYFP and 6-OHDA + eYFP group. Scale bar: 200 μm **(D)**. *p < 0.05.

In this experiment there were four groups. One group that received saline in the striatum and eYFP in the STN (*n* = 6). This is the saline +eYFP group and acts a control to assess changes in 6-OHDA rats. The other three groups received 6-OHDA and either eYFP (*n* = 5) ChR2 (*n* = 8) or NpHR3.0 (*n* = 8) in the STN (Figure 3A). The placements of optic cannula and virus expression are shown in Figure 3B. The extent of dopamine neuronal loss was assessed after behavioural studies. The number of TH immunoreactive neurons were significantly reduced in the 6-OHDA lesion groups compared to saline +eYFP group, *F*_(3,22)_ = 18.398, *p* ≤ 0.001 (Figures 3C,D).

**FIGURE 4:**
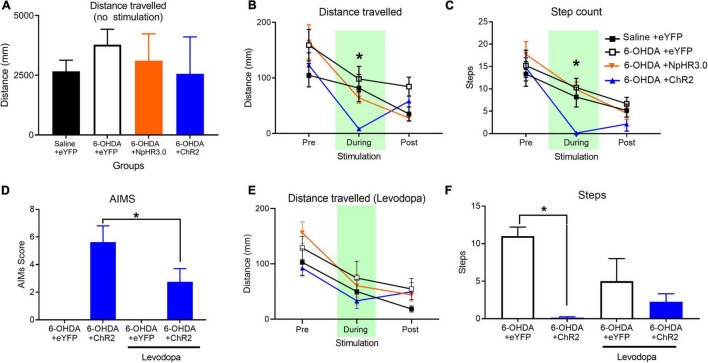
Changes in locomotor in PD rodent model upon STN stimulation and Levodopa administration. No significant effect on groups on distance travelled during no optogenetic manipulation **(A)**. A 30 s open field test involving 10 s pre-, during and post-stimulation periods demonstrated a significant decline in distance **(B)** and step count **(C)**. Levodopa administration significantly reduced abnormal involuntary movement (AIMS) **(D)** rescues impact on distance travelled **(E)** significantly lower step count **(F)**. Data is expressed as mean ± SEM, *p < 0.05.

All groups were assessed for locomotor changes in the open field without stimulation for 20 min. There were no differences between groups for ambulatory distance travelled *F*_(3,22)_ = 1.206, *p* = 0.331 (Figure 4A). To assess the impact on locomotor upon STN stimulation, the following locomotor assay was segmented into three phases; pre-stimulation, stimulation, and post-stimulation phase. We observed that optogenetic stimulation of the PD +ChR2 induced severe motor symptoms of abnormal involuntary movements (AIMs) including twisting of the neck, tremor of the forelimbs and jaw, and inability to move voluntarily. Unlike the reduced motor responses observed during STN optogenetic activation in non-lesioned rats, under dopamine depletion activation of the STN induced severe motor symptoms including (see Supplementary Videos). Hence optogenetic manipulation period was reduced to 10 s in contrast to 1 min in the presence of dopamine to reduce the period of adverse impact on the rats. We found this time sufficient to detect significant deficits (see Supplementary Videos). For distance travelled, there was no difference between all groups during pre-stimulation, *F*_(3,22)_ = 1.178, *p* = 0.341 and post-stimulation phase, *F*_(3,22)_ = 1.555, *p* = 0.229. There was significant difference during optogenetic stimulation, *F*_(3,22)_ = 7.156, *p* = 0.002. The 6-OHDA + ChR2 group had significantly reduced movement compared other groups, *p* < 0.02, Figure 4B. For step counts, there was no difference between all group during pre-stimulation, *F*_(3,22)_ = 0.680, *p* = 0.573 but there was significant decrease during stimulation, *F*_(3,22)_ = 14.684, *p* < 0.001 and post-stimulation phase, *F*_(3,22)_ = 3.141, *p* = 0.046, Figure 4C. *Post hoc* analysis show significant difference only the 6-OHDA + ChR2 group during stimulation. In the post-stimulation phase, significantly decrease was detected between 6-OHDA + eYFP and the 6-OHDA + ChR2 group, *p* = 0.035.

### Experiment 4: Application of Levodopa Rescues Motor Deficits Induced by Subthalamic Nucleus Excitation in 6-Hydroxydopamine-Lesioned Rats

The prominent motor deficits observed in only the group with dopamine loss and STN activation led us to consider the “two hit hypotheses” for PD motor symptoms where the loss of dopamine along with increase in STN activity leads to the motor dysfunction. To test this hypothesis, we thought to supplement all groups with levodopa prior to stimulating the STN. For statistical analysis paired samples *t*-test analysis was applied as AIMs was detected in the one group. Analysis demonstrated that in the presence of levodopa there was significant improvement of AIMs in the ChR2 group, *t*_(7)_ = 3.451, *p* = 0.011, Figure 4D. There was also no significant difference in distance travelled during pre-stimulation, stimulation or post-stimulation phases, *F*_(3,20)_ < 1.706, *p* > 0.05, Figure 4E. There was a significant reduction in number of steps during STN excitation between the control and ChR2 group, *F*_(1,11)_ = 129.647, *p* < 0.001. Administration of levodopa rescued the motor deficits, leading to no group difference in steps between control and ChR2 with levodopa, *F*_(1,11)_ = 1.034, *p* = 0.331, Figure 4F.

## Discussion

Therapeutical targeting of the STN *via* lesion or deep brain stimulation improves motor symptoms in PD patients (Limousin et al., 1995). A major interest in the field is to decipher the pivotal role of STN in motor control and dysfunction. To address these fundamental questions, we measured motor function while using optogenetics to selectively activate and inhibit STN neurons under normal and dopamine depleted conditions.

We found that direct inhibition of STN neurons increases locomotion, consistent with its position in striatal indirect pathway. Conversely, direct excitation of STN decreased movement in rats (Figures 2D–H). Similar results have been observed in mice whereby optogenetic inhibition of STN neurons induces hyperlocomotion (Schweizer et al., 2014; Guillaumin et al., 2020; Heston et al., 2020; Pamukcu et al., 2020) and direct optogenetic excitation of the STN reduces specific locomotion tasks (Guillaumin et al., 2020). These results suggest that STN neuronal activity is capable of bidirectional motor regulation within the indirect pathway and suggests that STN-DBS has an inhibitory effect on STN glutamatergic neurons.

Clinical studies show STN-DBS may impact non-motor behaviour (Bjerknes et al., 2021; Voruz et al., 2022). STN optogenetic stimulation or inhibition had no effect on the real time place preference assay (Figure 2I). The null effect of STN optogenetic manipulation on non-motor behaviour maybe driven using CaMKII promoter used in our study to selectively express in excitatory glutamatergic neurons (Egashira et al., 2018; Hoshino et al., 2021). Application of specific promotors such as CaMKII promoter limits manipulation of excitatory glutamatergic neurons and not effecting inhibitory neurons or passing fibers (Gradinaru et al., 2009). DBS electrical stimulation in the STN also impacts the local network, including stimulation of some STN neurons, inhibition of other STN neurons, and activation of passing fibres in the STN (Bosch et al., 2011; Dvorzhak et al., 2013). Conditional reduction of vGLUT2 neurons in the STN induces hyperlocomotion in mice without impacting limbic and cognitive functions (Schweizer et al., 2014). Together with others, our study reveals the potential of cell specific targeting of STN neurons with optogenetics technology for targeting specific behaviours akin to electrical DBS.

Dopamine loss is a pathological hallmark of PD. To better understand the relationship between STN activity and motor function in PD we examined the effect of STN modulation in rats with 6-OHDA lesions. Optogenetic activation of striatal neurons (non-selective) in unilateral 6-OHDA show similar dyskinesia phenotype (Hernández et al., 2017). Here we demonstrate temporally that the increased activity of the STN with the application of optogenetics and absence of dopamine using pharmacological manipulation severely impact motor control.

Subthalamic nucleus excitation-induced motor dysfunction was dependent on the loss of dopamine as augmentation of dopamine transmission by Levodopa attenuated the STN excitation-induced motor deficits. It’s unclear whether Levodopa acts on STN neurons directly or indirectly. STN neurons expresses dopamine D1 and D2 receptors (Boyson et al., 1986; Hassani et al., 1997) and focal application of dopamine agonists into the STN reduces neuronal excitability in the STN (Hassani and Féger, 1999). Our study demonstrates that STN motor function is impacted by the absences and presence of dopamine (Figure 4).

### Methodological Considerations

A caveat of our experimental model is the sub-sequential effect of pharmacological manipulation of dopamine. It is possible that the loss of dopamine alters STN activity (Benazzouz et al., 2000). However, we included two other groups with dopamine depletion [the control group (6-OHDA + eYFP) and inhibition (6-OHDA + NpHR3.0) group] which did not display motor deficits. Albeit of the initial brain region of aberration, we show evidence that both STN and dopamine abnormalities together lead to motor deficits. This study leads to the key question of does STN pathology precede dopamine loss or if loss of dopamine induces alteration in STN activity.

## Conclusion

Here we show the key neural pathologies of PD motor symptoms is inclusive of dopamine depletion and over activity of the STN. Serendipitously, we show a novel and robust PD animal model for assessing pharmacotherapies for PD motor symptoms. For example, the therapeutic effects of levodopa in this study sets the platform to assess other pharmacological agents for PD treatment. The pathology is temporal and reversible upon optical stimulation. The combination of the traditionally applied neurotoxin rodent model with optogenetics provides relevant rodent model to for Parkinson’s motor symptoms. This model incorporates both hallmarks of PD pathology, setting a screening platform for future studies targeting STN directed treatments. Moreover, demonstrating optogenetics approach in characterizing the pathological features of PD, we can therefore develop new and improved therapeutic strategies to target STN dysfunction. Our findings demonstrate that dopamine loss and STN over activity are key features of PD motor symptoms. This draws insight to the underlying causes of PD motor symptoms, highlighting that while the loss of dopamine is the hallmark of PD, the STN is also an integral component of PD pathology and treatment.

## Data Availability Statement

The raw data supporting the conclusions of this article will be made available by the authors, without undue reservation.

## Ethics Statement

The study involving animals was reviewed and approved by the UNSW Animal Care and Ethics Committee. All studies were performed in accordance with the Animal Research Act 1985 (NSW), under the guidelines of the National Health and Medical Research Council Code for the Care and Use of Animals for Scientific Purposes in Australia (2013).

## Author Contributions

CX and AAP conducted behavioural and immunohistological experiments. JP carried out all electrophysiological experiments. All authors contributed to the preparation of the manuscript, contributed to the article, and approved the submitted version.

## Conflict of Interest

The authors declare that the research was conducted in the absence of any commercial or financial relationships that could be construed as a potential conflict of interest.

## Publisher’s Note

All claims expressed in this article are solely those of the authors and do not necessarily represent those of their affiliated organizations, or those of the publisher, the editors and the reviewers. Any product that may be evaluated in this article, or claim that may be made by its manufacturer, is not guaranteed or endorsed by the publisher.

## Supplementary Material

Supplementary Video 1 6-OHDA +NpHR3.0: Optic stimulation at 625 nm of the subthalamic nucleus (STN) with CaMKII- NpHR3.0 opsin does not affect motor performance in 6-OHDA lesioned rats.

Supplementary Video 2 6-OHDA +ChR2: Optic stimulation at 470 nm of the subthalamic nucleus (STN) with CaMKII-ChR2-eYFP opsin evoked abnormal involuntary motor performance in 6-OHDA lesioned rats.
